# Tissue engineering strategies for human hair follicle regeneration: How far from a hairy goal?

**DOI:** 10.1002/sctm.19-0301

**Published:** 2019-12-26

**Authors:** Ana Rita Castro, Elsa Logarinho

**Affiliations:** ^1^ Aging and Aneuploidy Group IBMC, Instituto de Biologia Molecular e Celular Porto Portugal; ^2^ i3S, Instituto de Investigação e Inovação em Saúde, Universidade do Porto Porto Portugal; ^3^ Programa Doutoral em Engenharia Biomédica, Faculdade de Engenharia Universidade do Porto Porto Portugal; ^4^ Saúde Viável – Clínica de Microtransplante Capilar Porto Portugal

**Keywords:** alopecia, hair follicle, hair regeneration, stem cell, tissue engineering

## Abstract

The demand for an efficient therapy for alopecia disease has fueled the hair research field in recent decades. However, despite significant improvements in the knowledge of key processes of hair follicle biology such as genesis and cycling, translation into hair follicle replacement therapies has not occurred. Great expectation has been recently put on hair follicle bioengineering, which is based on the development of fully functional hair follicles with cycling activity from an expanded population of hair‐inductive (trichogenic) cells. Most bioengineering approaches focus on in vitro reconstruction of folliculogenesis by manipulating key regulatory molecular/physical features of hair follicle growth/cycling in vivo. Despite their great potential, no cell‐based product is clinically available for hair regeneration therapy to date. This is mainly due to demanding issues that still hinder the functionality of cultured human hair cells. The present review comprehensively compares emergent strategies using different cell sources and tissue engineering approaches, aiming to successfully achieve a clinical cure for hair loss. The hurdles of these strategies are discussed, as well as the future directions to overcome the obstacles and fulfill the promise of a “hairy” feat.

## Graphical abstract

Steps toward human hair follicle regeneration. Cell isolation: cell sources for bioengineering can be follicular (bulge stem cells, dermal papilla, and dermal sheath cells) and non‐follicular (keratinocytes, skin‐derived progenitors, and mesenchymal stem cells). Cell expansion: mesenchymal and epithelial cell sources are cultured in vitro. Bioengineering: cell clustering in 3D instructive hair bulbs. Implantation: bulbs could generate functional hair follicles.

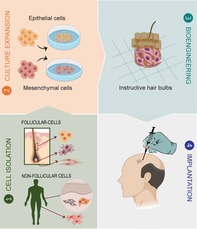

**Significance statement**

Hair loss (alopecia) affects a growing number of people worldwide. Limited efficacy and side effects of current pharmacological and surgical treatments have fostered the search for alternative therapeutic solutions. Great expectation has been recently put on hair follicle bioengineering, which is based on the development of functional hair follicles from an expanded population of hair‐inductive cells. However, human follicle neogenesis resorting to patient's cells was not successfully achieved yet. Based on recent advances in the field, this review on cell‐based hair follicle tissue engineering systematically compiles the emerging strategies while disclosing the hurdles that still limit translation into the clinics.

## 1 INTRODUCTION

Hair loss (alopecia) is a disease that affects a growing number of people worldwide and impacts individuals' physical, psychological, and social well‐being.^1^ Patients with hair disorders suffer from emotional stress, embarrassment, and depression that severely compromise their life quality.^2^ Up to date, treatments include pharmacological and surgical (autologous hair transplant) interventions. Although hair restoration surgery is nowadays the most effective method, donor hair follicles (HFs) scarcity is often its major limitation.^3^ Besides, pharmacological treatments still not fully satisfy the patient's needs and entail drastic side effects.^4^ Thus, the limited efficacy and possible side effects of the current treatments have fostered the search for alternative therapeutic solutions, capable of generating unlimited number of HFs de novo. Noteworthy, stem cell‐based tissue engineering is emerging as the most thriving approach, aiming to reconstruct HFs in vitro to replace lost or damaged HFs as a consequence of disease, injury, or aging. HF bioengineering approaches are based on the accumulated knowledge on reciprocal epithelial‐mesenchymal (EM) interactions controlling embryonic organogenesis and postnatal HF cyclic growth. However, despite recent progress in the field, clinical applications of tissue engineering strategies for hair loss are still missing. Neogenesis of human follicles derived from cultured HF dermal cells has not been successfully achieved yet.

This review focus on the research approaches being developed to tackle the major limitations of human HF bioengineering, namely the loss of cellular function following in vitro HF cells expansion, the loss of in vivo tissue context/architecture, and the reconstruction of autologous functional HF germs for clinical procedures.

## 2 HF MORPHOGENESIS AND CYCLING: STEM CELL POPULATIONS

HF is a mini‐organ that forms during embryonic skin development. Its functional and cycling activities rely on a coordinated communication between the different cell populations from epithelial, mesenchymal, and neural crest stem cell origin,^5^ which additionally regulates adult skin homeostasis and wound repair.^6^, ^7^ Therefore, understanding the HF anatomy, as well as the stem‐cell populations operating during postnatal cyclic regeneration, is crucial for tissue engineering‐based solutions.

**Figure 1:**
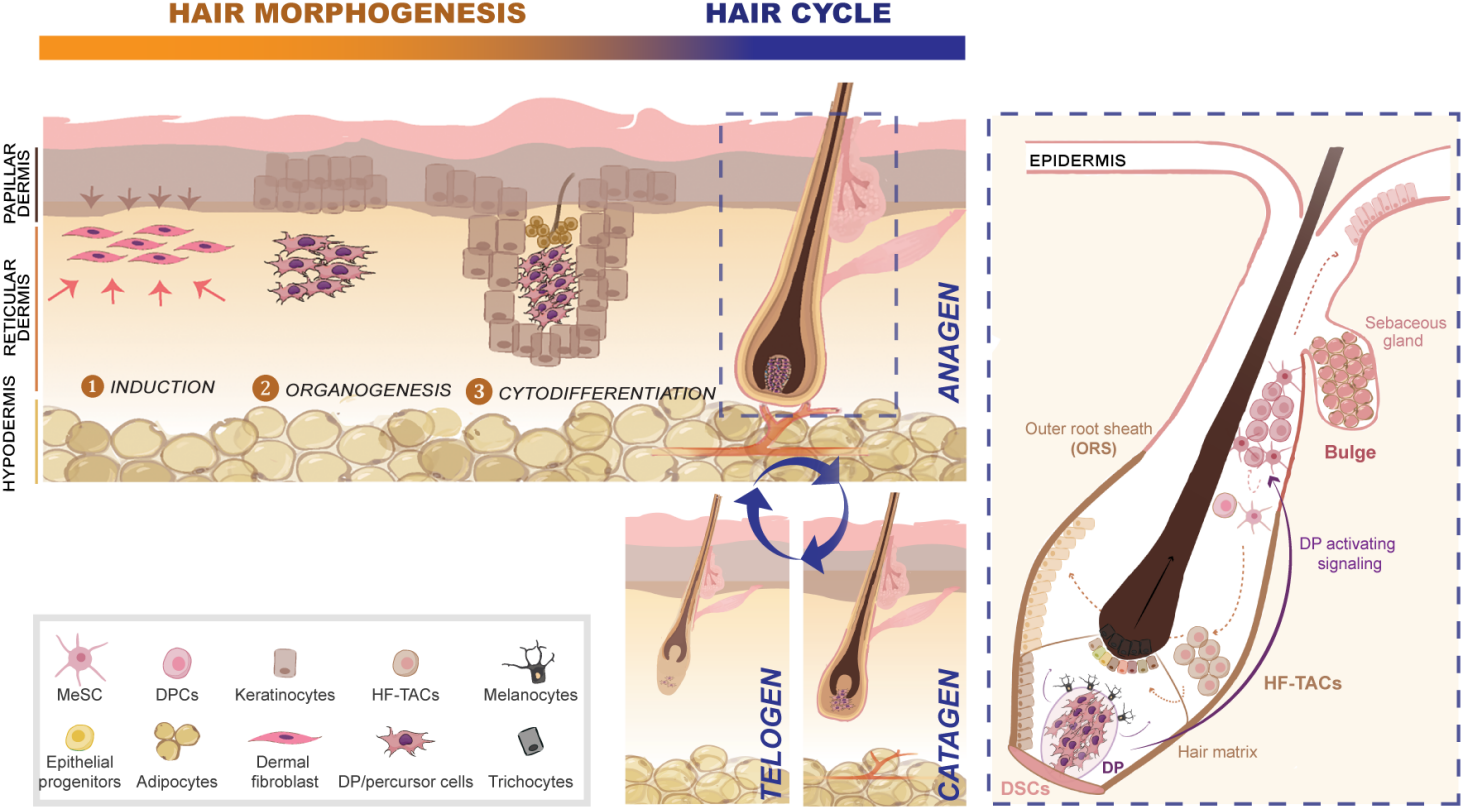
Cell population dynamics during hair follicle (HF) morphogenesis and cycling stages. Interactions between epithelial and mesenchymal cell populations in the skin determine the embryonic morphogenetic stages of induction, organogenesis and cytodifferentiation, as well as the postnatal hair cycling stages of anagen, catagen, and telogen. Inset on the right depicts the complex cell population dynamics operating during anagen. Bulge quiescent hair follicle stem cells (HFSCs) are activated by dermal papilla (DP) stimulatory signals to proliferate and generate HF‐TACs (HF‐TACs). HF‐TACs migrate out of the bulge and differentiate either in outer root sheath (ORS) or epithermal progenitors. HF‐TACs that migrate into the hair matrix give rise to several differentiated epithelial cell lineages (in different colors) that directly contribute to hair growth. Besides HFSCs, melanocyte stem cells (MeSC) in the bulge give rise to differentiated melanocytes that fuel pigment granules to the adjacent differentiating cells. Dashed lines represent cell trajectories and solid lines represent molecular signaling

Follicular dermal stem cells exist in the dermis (skin‐derived precursors, SKP) able to regenerate dermal sheath (DS), and populate the dermal papilla (DP) at every growth cycle.^8^ Both DS and DP comprise mesenchymal cells with multi‐lineage differentiation capacity.^9^ In the mature HF, the DP is adjoined to connective tissue sheath (DS), together forming the dermal component of the mature HF^10^ (Figure 1). The DP is thought to be a master regulator of HF cycling, which consists in serial phases of growth (anagen), apoptotic‐driven regression (catagen), and rest (telogen).^11^ On the human scalp, anagen lasts 1‐6 years and it involves the complete regeneration of the cycling portion of the HF (Figure 1). At the telogen‐to‐anagen transition, DP stimulates epithelial hair follicle stem cells (HFSC) from the bulge region, which are adult multipotent cells holding self‐renewal capability and kept quiescent in their niche surrounded by the sebaceous gland (SG) in the outer root sheath.^12^, ^13^ When DP stimulatory signaling overcomes the threshold imposed by the inhibitory bulge microenvironment,^14^ HFSCs divide generating a new pool of progenitors at the bulge base called the secondary germ cells,^15^ which survive catagen‐driven apoptosis.^16^ These primed hair germ cells migrate to the bulb, while expanding and differentiating into transit‐amplifying cells (HF‐TACs) that attach to the basement membrane surrounding the DP lower half. HF‐TACs likely sit in place throughout much of anagen to fuel HF growth by differentiating into eight distinct epithelial lineages (eg, shaft, inner root sheath, and companion layer cells) and SGs^17^, ^18^, ^19^ (Figure 1).

In addition to HFSCs, melanocyte stem cells also reside in the bulge. During anagen, they are coordinately activated with HFSCs to generate mature melanocytes that produce and distribute pigment granules to the adjacent differentiating cells to form pigmented hair fibers.^20^ Catagen phase can last between 4 and 6 weeks, where keratinocytes and melanocytes undergo apoptotic processes. This apoptotic‐driven regression causes DP to move upward, bringing it closer to the epithelial bulge.^21^ Following complete regression, HF enters a quiescent phase (telogen), which can last several months. The replacement of the old hair shaft fiber by the forming club fiber at the end of telogen is called “exogen.”^22^ Finally, HF macroenvironment also encompasses adipocyte tissue containing adipose‐derived stem cells in close commitment with hair growth regulation, as patented by increased adipose tissue thickness in anagen.^6^

Altogether, the DP, differentiated epithelial cells and the hair matrix constitute the cycling portion of the postnatal HF, being actively renewed each HF cycle. Conversely, the upper portion of the follicle (including bulge, SG appendage, and the infundibular epidermis) constitute the permanent portion of the postnatal HF, being formed during embryonic development and kept throughout life.^23^, ^24^ Any aberrant signaling affecting the communication between mesenchymal DP and the surrounding epithelial cells will disrupt the hair cyclic regeneration during postnatal life.

## 3 HF BIOENGINEERING: CELL SOURCES AND CHALLENGES

Stem cell‐based regenerative medicine is emerging as the most thriving approach for hair loss treatment by holding the potential of HF cloning, that is, the production of bioengineered instructive germs from human HF cells expanded in vitro to generating fully functional HFs upon transplant into the patient's bald scalp. Rationally, such a regenerative therapy may only be possible if combining receptive‐epithelial and inductive‐mesenchymal populations to mimic the well‐orchestrated interactions controlling lifelong HF cycles, which are deeply affected during hair loss.^25^ Ideally, a cell‐based regenerative medicine therapy would be autologous, that is, resort to patients' cells derived from small amounts of tissue biopsies (eg, HF punch). Thus, researchers in the field have been mainly focused on developing therapeutic bioengineering solutions using dissociated HFSCs and DP cells (DPCs) isolated from HF biopsies. HFSCs and DPCs retrieved from nonbalding scalp follicles should first be expanded in culture to produce bioengineered structures in vitro with hair regenerative potential. Still, an allogeneic cell source could be alternatively used for HF regenerative therapy. Two decades ago, transgender transplantation of microdissected DP and DS was shown to successfully induce HFs.^26^ This study not only pointed to the need of an inductive dermal component for HF regeneration, but it also disclosed the possibility of using an allogeneic cell source for therapy. Indeed, HF proved to be an immune‐privileged site, as it does not express MHC (major histocompatibility complex) class I antigens.^27^, ^28^ Regardless of autologous vs allogeneic therapy, the relevance of HFSCs, and mainly DPCs, on tissue‐engineering approaches for treating alopecia has been the focus of intensive research over the last decade.

### 3.1 The relevance of the DP and strategies tackling its hair‐inductive potential

Seminal studies demonstrated that papillae isolated from rat, guinea‐pig vibrissa, but also human, could induce HF formation when implanted into a recipient non‐hairy skin.^29^, ^30^ These experiments demonstrated that DP, unlike dermal fibroblasts, can reprogram non‐hairy epidermis to a follicular fate. Later on, several groups demonstrated hair regeneration in immunodeficient mice skin when combining different populations of dissociated cells,^31^ including DP and DS,^32^ but also freshly isolated epithelial bulge cells.^33^ More recently, ablation of DPCs unequivocally demonstrated its pivotal role on hair growth but not on epithelial HF maintenance.^13^ Moreover, the generation of rodent pelage by transplantation of dissociated DPCs has been possible in a variety of different approaches, as revised in Ohyama et al.^34^ However, inducing human HF‐like structures in nude mice only proved possible when human DPCs were combined with epidermal component,^35^, ^36^, ^37^ clearly indicating the need for EM interactions. Thus, a successful bioengineering solution will require an available amount of competent epithelial and inductive DPCs.

DP in vitro cell expansion is inevitable toward the development of a clinically relevant tissue engineering‐based solution for HF regeneration. However, two major technical burdens have limited the attainment of human trichogenic DPCs. In contrast to mouse, isolation of human DPCs requires DP microdissection from HF punches, as enzymatic digestion with trypsin and collagenase inefficiently works to generate single cells for FACS‐sorting, and robust cell surface markers (as, eg, CD133 in the mouse) remain to be defined.^34^ Moreover, enzymatic digestion would deprive DP of its natural and distinctive extracellular matrix microenvironment, which is essential for hair inductive properties. This is also a caveat of in vitro expansion, particularly under regular culture conditions lacking key environmental cues.^38^, ^39^ Considering the relevance of DP for HF cycle, massive effort has been made on finding out an in vitro expansion procedure for human DPCs that preserves, or alternatively rescues, their hair inductive ability. One approach consists in coculturing DPCs with keratinocytes or keratinocyte‐conditioned medium, shown to provide DPCs with molecular cues involved in EM crosstalk during hair growth.^35^, ^40^ Also, distinct players in this signaling crosstalk (including Wnt, BMP and FGF) were reported to prolong the hair‐inducing ability of cultured DPCs.^38^, ^39^, ^41^, ^42^, ^43^ More recently, pharmacological modulation of JAK–STAT signaling pathway was shown to improve cultured human DPCs’ inductivity.^44^ Another strategy to reproduce native DP trichogenicity is the establishment of three‐dimensional (3D) sphere cultures, which has been pointed out as one of the most effective means to restore intact anagenic DP transcriptional signature.^45^ The 3D‐spheroid culture help DPCs to aggregate, thereby reestablishing cell‐cell contacts, a crucial feature of in vivo DP.^46^ In fact, sphere formation was shown to increase hair‐inductive ability in both cultured human DPCs and multipotent dermal progenitors (SKPs).^47^, ^48^, ^49^

Considering the undoubted contribution of 3D culture for DPCs' fitness, numerous studies have explored the contribution of biomaterial‐based strategies to support DP hair inductive behavior in vitro.^43^, ^50^ Biomaterials have been employed either (a) as supportive scaffold with known stiffness for 3D cell culture^51^, ^52^; or (b) as supportive matrix for the encapsulation of dissociated cells.^37^, ^53^ Recently, human placenta extracellular matrix hydrogel was used for DP sphere culture and shown to restore hair‐inductive potential of high‐passage DPCs.^54^ Moreover, 3D culture has been combined with different top‐up modulations to boost cultured DPCs' inductivity. Importantly, by combining Wnt signaling pharmacological activation of DPCs with 3D spheroid culture was recently shown to enhance de novo HF formation in a reconstituted human skin grafted into nude mice model.^55^

### 3.2 The requirement of the HFSC epithelial component

Bulge HFSCs are the most prominent stem cell population actively contributing to HF regeneration in vivo by replenishing different epithelial lineages.^7^, ^56^ Although their depletion convey in HF loss,^7^ in the most common pathological hair loss conditions, that is, alopecia areata (AA) and androgenic alopecia (AGA), HFSCs are preserved (but not DP cells).^57^ In contrast, aging‐driven loss of hair regenerative capacity is associated with terminal differentiation and transepidermal clearance of HFSCs.^58^ Thus, from an in vitro tissue engineering perspective, mimicking in vivo contribution of both HFSC and DP seems critical to establish a successful HF regeneration strategy. Accordingly, several studies evidenced a boost of HF induction when combining the epithelial component (HFSCs ± keratinocytes) with the inductive DPCs within engineered germs.^59^, ^60^ Even though, bulge‐specific cell molecular markers allow direct HFSC isolation from mouse and human HF^33^, ^61^ —as opposed to human DPCs—HFSCs have poor proliferative ability and tend to differentiate during culture passaging.^62^ Therefore, committed progenitors or keratinocyte precursors are being alternatively used as an epithelial component for HF tissue engineering.^63^ Actually, different EM combinations have been tried to successfully reconstruct folliculogenesis in vitro. To date, fully functional HF reconstruction in vitro was only achieved using mouse embryonic skin cells.^37^, ^64^ Several studies have reported improved HF neogenic potential either by using cells with higher differentiation potential, namely adult HFCS, neonatal keratinocytes and embryonic cells,^37^, ^65^ or by coupling human and mouse cells to form chimeric HFs (eg, human mesenchymal inductive cells and rodent epithelial keratinocytes).^66^, ^67^ As expected, such cellular constructs evidence greater HF inductive potential when implanted into a mouse skin. Although interesting from a mechanistic perspective, those studies are far from reproducing a postnatal human HF regeneration. Conversely, they may bewilder the layman community by suggesting successful fully functional HF regeneration (ie, HF with cycling activity, hair shaft grow, and stem cell renewal) by non‐translational approaches. Importantly, as most studies have used rodent cell‐based assays (Table 1), the actual contribution HFSCs for human HF bioengineering needs to be further validated. Although challenging, some researchers are now committed to establish in vitro human post‐natal cell‐based HF germs.^43^, ^55^

**Table 1:** Summary of tissue engineering approaches tested with HF regeneration outcome

Origin	Mesenchymal cell type	Epithelial cell type	In vitro approach	In vivo approach	outcome	Ref
Mouse	SKPs	Neonatal epidermal KTs	TSA treatment in SKP aggregates	Transplantation in nude mice excisional wounds	HF neogenesis	68
	Adult dermal fibroblasts	Dorsal skin epidermal KTs	Treatment with embryonic skin extract	Patch assay in nude mice full thickness wounds	HF neogenesis	69
	Adult vibrissa DPCs	Adult vibrissa HFSCs	3D culture	Intracutaneous transplantation of HF germs in nude mice	HF formation	70
		Neonatal foreskin epidermal KTs	PRP‐based bioactive 3D scaffolds	Mini chamber assay in nude mice	HF formation	71
	Neonatal dermal progenitors	Neonatal epidermal progenitors	3D organoid	Transplantation into nude mice	HF formation	72
	Neonatal dermal cells	Neonatal epidermal cells	3D coculture in collagen scaffold		HF formation	50
	iPSCs‐derived MSCs	iPSCs‐derived epithelial stem cells	Bioengineered 3D integumentary organ system	Embryonic body transplantation into nude mice	Functional HF regeneration	73
	Embryonic back skin follicle‐derived mesenchymal cells	Embryonic back skin follicle‐derived epithelial cells	Bioengineered HF germ using organ germ method	Intradermal transplantation into nude mice	Functional HF regeneration	37
Mouse and human	Mouse neonatal dermal cells	hiPSC‐derived bulge stem cells	‐	Chamber assay in nude mice	HF formation	74
	Mouse DP‐enriched cells	Human neonatal foreskin epidermal KTs	Mixed cell suspension	Chamber assay in nude mice	HF formation	66
	DP‐like cells derived from neural crest hESCs	Mouse neonatal epidermal KTs		Subcutaneous injection in nude mice	HF formation	75
	Mouse whisker DPCs	Human epidermal KTs	Cell‐matrix composites	Graft skin composite in SCID mice	HF neogenesis in regenerated skin tissues	67
	Human DPCs	Mouse embryonic epithelial cells	DP encapsulation in collagen‐enriched microgel	Patch assay‐ transplanted slab of nude mouse skin	Microtissue fabrication with increased hair regeneration	65
Human	Intact DP	Bulge derived epithelial cells	Bioengineered HF germ using organ germ method	Intradermal transplantation in nude mice	Human bioengineered hair follicle	37
	Scalp HF DPCs	Neonatal epidermal KTs	DP aggregates in collagen I 3D bioprinting molds	Engraftment into SCID mice	Generation of Human HF within human skin constructs	36
		Adult epidermal KTs	Hanging drop culture for 3D spheroids	‐	Spontaneous cell sorting within mixed aggregates	43
		HF KTs and HFSCs	3D DP organoid within a gelatin hydrogel	‐	Relevance of EMI in HF	60
		Melanocytes and ORS KTs	Engineered microfollicles	‐	HF fibers similar to vellus hair	76
	Scalp HF DPCs and dermal fibroblasts	Fetal epidermal KTs	Wnt signaling activation in DP spheroid culture	Reconstructed human skin assay grafted into nude‐SCID mouse	Functional HF formation	55
	Adult SKPs	Adult epidermal SCs	Cells mixed in a hydrogel	Transplantation in nude mice excision wound	HF and SG formation	77

Abbreviations: DPCs, dermal papilla cells; EMI, epithelial‐mesenchymal interaction; HFSCs, hair follicle stem cells; KTs, keratinocytes; ORS, outer root sheath; PRP, platelet‐rich plasma; SCs, stem cells; SG, sweat gland; SKPs, skin derived progenitors; TSA, trichostatin A.

### 3.3 Non‐follicular cell sources for HF bioengineering

While isolation of HFSCs and DPCs seems a promising autologous cell source for human HF regenerative therapy, non‐follicular cell populations have been also considered, especially when donor hair scarcity is an issue. Due to their similarity with DPCs, SKPs have been alleged as an autologous multipotent cell source for HF regeneration.^78^ They have proved clinically relevant for stem cell‐replacement strategies, particularly in central nervous system and spinal cord injury models.^79^ Also, SKPs have been extensively studied for its trichogenic potential in mouse skin.^68^, ^77^ Reconstruction of functional HF and SGs in nude mice excisional wound has been achieved following implant of spheroid structures containing epidermal stem cells and SKPs in a hydrogel.^77^ However, hair inductive potential of this precursor population in the human remains unclear, in addition to its challenging isolation from human dermis.

To overcome the constraints of cell population scarcity and/or technical isolation, non‐autologous reprogramed pluripotent stem cells have been considered as these represent an inexhaustible source of cells with multi‐lineage differentiation ability.^80^ To date, several groups have already attempted HF regeneration using human‐induced pluripotent stem cells (hiPSCs).^73^, ^74^, ^75^ Differentiation of hiPSCs obtained from non‐hair cell sources can be used to generate both DPCs and HFSCs, or simply an inductive dermal and receptive epidermal cell population. Indeed, hiPSCs may enable HF bioengineering through a 3D integumentary organ system in vitro. In these systems, iPSCs‐based organ germs were created to stimulate bioengineered organ development in vivo through reciprocal EM interactions.^73^ Nevertheless, the use of iPSCs for human therapeutic purposes is still controversial due to safety, namely viral integration into the genome and risk of teratoma formation.^81^ Stringent safety requirements will need to be satisfied toward cGMP manufacturing and commercialization of hiPSC‐derived therapeutic products prior to their clinical application.^82^ Also, human embryonic stem cells (hESCs) have been tested for differentiation into HF cell lineages. Recently, a two‐step procedure reported the derivation of hESCs into neural crest cells and then hair inductive DP‐like cells.^75^ Lastly, recent studies described the transformation of human dermal fibroblasts into hair‐inducing cells with HF regenerative competence.^67^, ^69^

**Figure 2:**
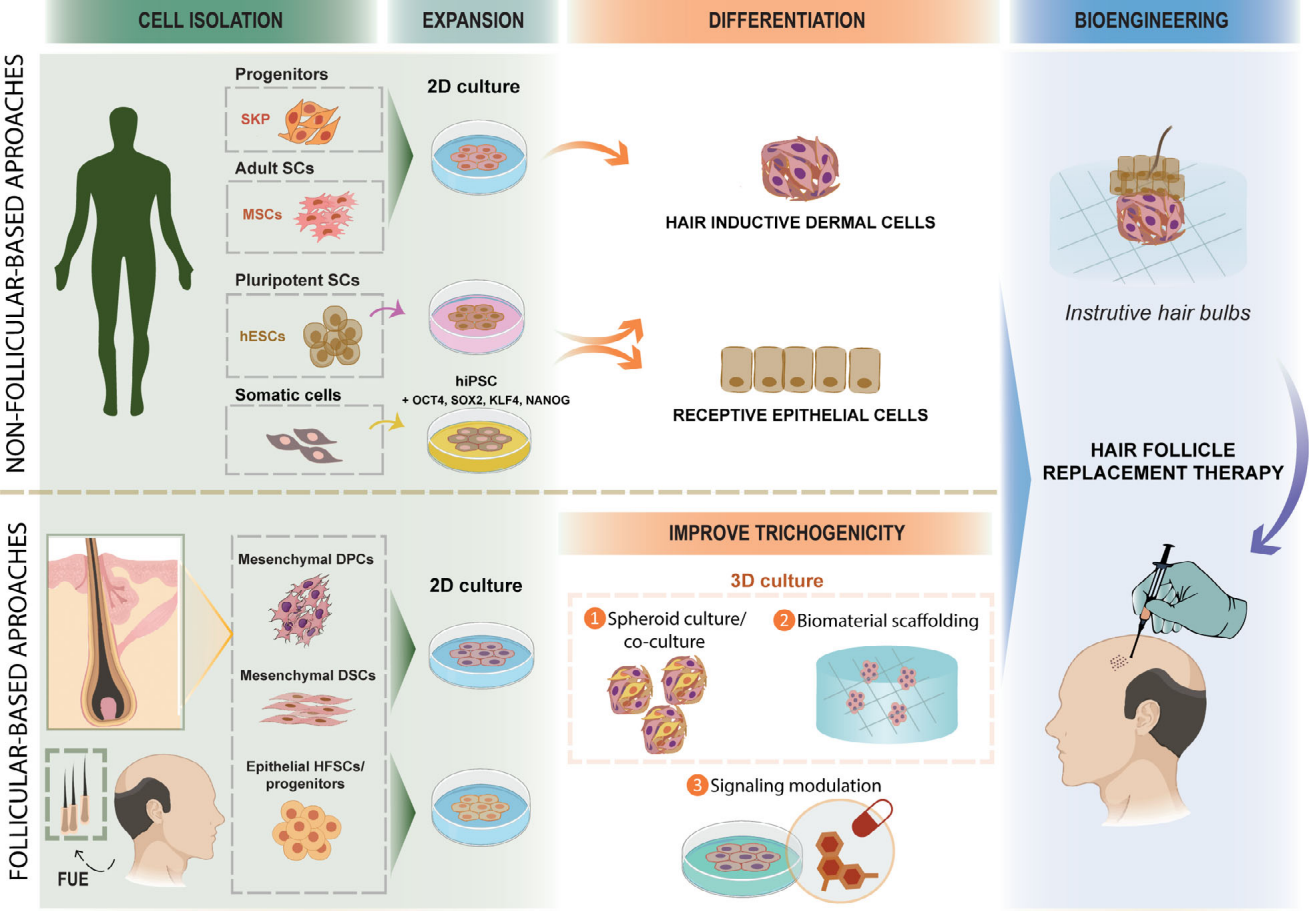
Prospective cell‐based strategies for hair follicles (HF) regenerative therapy. Different cell sources are being explored for HF tissue engineering. Non‐follicular cell sources (top left): skin derived progenitors (SKPs) from human skin, which are similar to dermal papilla cells (DPCs); adult stem cells (eg, isolated from bone marrow, MSC); and pluripotent stem cells (embryonic/ESC or induced/iPSC with Yamanaka factors), which may be differentiated into a mesenchymal inductive population (akin to follicular DP) or a receptive epithelial population. Follicular cell sources (bottom left): DPCs and DSCs mesenchymal inductive; and bulge hair follicle stem cell (HFSC) epithelial receptive. Considering the negative effects of in vitro follicular cell culture expansion, signaling modulation, 3D culture, biomaterial‐based culture, or a combination of these approaches may be used to restore cell's trichogenicity. By combining epithelial and mesenchymal components, engineered instructive mini‐bulbs can be obtained in vitro for a successful tissue engineering solution able to generate mature and functional HFs in the bald scalps; FUE, follicular unit excision

In sum, several cellular sources are currently being investigated for human HF bioengineering (Figure 2) that will deliver distinct HF equivalents in vitro with attractive potential to specific research/clinical applications (eg, preclinical models for drug efficacy testing and reconstructed skin models^76^, ^83^), even if do not answer the demand for functional/cycling/oriented bioengineered HF required for hair cloning.

## 4 REGENERATIVE MEDICINE THERAPY FOR HAIR LOSS: HOW FAR?

Considering that transferring isolated stem cells into a hostile microenvironment may encumber proper hair formation, different tissue engineering strategies have been designed to overcome this gap consisting either in: (a) biomaterial‐based platforms, including organic or inorganic matrices and scaffolds; (b) scaffold‐free microtissues, through the creation of multicellular spheroids or organoids; or more recently, (c) tissue bioprinting techniques.^84^ Using those different approaches, bioengineered instructive mini‐germs have been generated in vitro, which can be then transplanted into the host to produce HFs (Figure 2).

Inspired by HF embryonic development, a 3D organ germ method was proposed for HF bioengineering, consisting in providing a native‐like 3D environment and maximizing EM cell interaction to mimic hair organogenesis.^70^ Bioengineered follicle germs were shown to develop a correct structure when transplanted into the back skin of nude mice.^37^, ^70^ Additionally, two recent studies have achieved growth of mouse hair in vivo after transplantation of in vitro formed structures, either consisting of mouse adult DP and epidermal cells,^72^ or of mouse iPSCs,^73^ encapsulated in hydrogel matrices. Different artificial 3D microenvironments composed of silk‐gelatin,^60^ hyaluronic acid,^43^ and collagen^65^ were shown to improve hair germs for hair regenerative medicine. In a similar approach, human HF resembling vellus hair were recreated in vitro from a mixture of DP cells, keratinocytes and melanocytes in a collagen matrix, and named “microfollicles.”^76^ Presently, researchers are joining efforts to develop more complex structures that can mimic the DP tridimensional morphology while providing a spatiotemporal delivery of molecular cues needed for human hair morphogenesis. Such a combined approach may favor cellular interactions in vitro, hopefully guiding the development and differentiation of both epithelial and mesenchymal counterparts to form a mature HF. To this end, recent work disclosed an interesting approach using a bioactive scaffold based on platelet‐rich plasma that synergistically combines 3D culture environment with natural release of endogenous growth factors.^71^

Envisioning the large‐scale production of HF germs needed for a clinical setup, different innovative high‐throughput strategies have been conceived, using both mouse and human cells. 3D‐printing technology is currently being used in the hair research field to print 3D molds resembling HF microenvironment. Effective 3D‐printing of skin substitutes with human HFs has been recently reported.^36^ Additionally, custom‐designed array plates were produced to allow scalable fabrication of inductive DP microtissues.^50^, ^65^

Finally, an emerging trend in HF research is the in vitro reconstruction of artificial hair‐bearing skin. Relevantly, Zhang et al were able to generate HFs from cultured mouse DP cells in de novo engineered skin model.^67^ Also, hair‐bearing human skin constructs were produced using innovative scaffolds that allow the development of properly oriented HFs.^36^

Despite the above‐mentioned progress in HF bioengineering, the reconstitution of a fully organized and functional human HF resorting to cultured human cells is still missing. A regenerative medicine therapy for human hair loss will only be successfully achieved when HF are formed de novo following implementation of in vitro bioengineered structures into the patient's bald scalp. Importantly, although from a scientific perspective studies have achieved and reported HF regeneration from human cells,^36^, ^76^ the caveats are whether (a) there is any mouse contribution in HF neogenesis from human bioengineered structures transplanted into mouse skin, and (b) human bioengineered structures will generate HF that besides growing/cycling also mimetic natural hair type and are responsive to physiological stimuli.

From a clinical perspective, an effective regenerative medicine would provide an autologous cell‐based bioengineered product able to cure hair loss without adverse side effects. Although promising, so far only hematopoietic stem cell‐based therapies have been implemented in the clinics. Moreover, significant limitations may further hamper an operational clinical solution for hair loss. First, bioengineered hair reconstruction will imply large‐scale production of cell‐based structures and the development of xeno‐free and well‐defined culture expansion media for clinical usage. Robust culture systems that allow stem cell expansion while maintaining their intrinsic properties are still missing. Second, even if generation of functional and cycling HF units is achieved, a huge gap still exists until the conception of a clinically relevant bioengineered product that responds to physiological stimuli (eg, neuronal stimuli) and aesthetic context (hair type, density, pigmentation, and orientation). For instance, larger bioengineered DPs could be required to generate thicker hair fibers, as DP size has been reported to impact on hair's diameter.^85^ Third, the low efficiency of organ induction, together with glitches in HF eruption and/or growth direction, may hinder the establishment of the effective therapy. Finally, from an economic perspective, a cost‐effective cellular expansion and in vitro cell‐based bioengineering for hair loss will be challenging. The establishment of a patient‐customized therapy will necessarily make it highly expensive.

Considering all the above‐mentioned pitfalls that the hair‐cloning premise has faced over the last decades, it is not surprising why hair rejuvenation (by stimulating existing follicles) has become the goal post for treating hair loss.

## 5 CONCLUSION

Comprehensive knowledge of HF morphogenesis and cyclic regenerative regulation, together with optimized protocols for HF/stem cells isolation and culturing have boosted the creation of a wide range of bioengineering solutions aiming to cure hair loss. However, future efforts are still needed to bridge such knowledge into an effective translational tissue engineering solution. Importantly, the successful development of in vitro engineered human HFs will certainly suit major biological applications far beyond hair loss cure. The conception of biologically improved skin replacement therapies (whose usage has been limited by the absence of HF), or even their application as a research model for skin drug development or cosmetic products testing, turn the HF bioengineering a knowhow seeker by several medical and pharmaceutical industries.

## CONFLICT OF INTEREST

The authors indicated no potential conflicts of interest.

## AUTHOR CONTRIBUTIONS

A.R.C., E.L.: conceptual design and manuscript writing.

## Data Availability

Data sharing is not applicable to this article as no new data were created or analyzed in this study.
